# Intestinal Failure: Epidemiology, Catheter-Related Sepsis and Challenges

**DOI:** 10.7759/cureus.16093

**Published:** 2021-07-01

**Authors:** Nikhil Nanjappa Ballanamada Appaiah, Kirsten Boyle, Daniel Rogers, James Stewart, Melanie Baker

**Affiliations:** 1 General Surgery, Basildon and Thurrock University Hospital, Basildon, GBR; 2 General and Colorectal Surgery, University Hospitals of Leicester, Leicester, GBR; 3 Gastroenterology, University Hospitals of Leicester, Leicester, GBR; 4 Nutrition Services, University Hospitals of Leicester, Leicester, GBR

**Keywords:** intestinal failure, parenteral nutrition, catheter related sepsis, through catheter antibiotics, gut rest

## Abstract

Background

The Leicestershire intestinal failure team (LIFT) service provides specialist intestinal failure care to patients in Leicestershire and surrounding counties.

Aims

To study the epidemiology of intestinal failure (IF) cases referred to the service and review outcomes particularly the indications and delivery of parenteral nutrition (PN). To review catheter-related sepsis (CRS) rates and identify areas for improvement.

Methods

Service data from January 2016 to November 2017 was analysed retrospectively. Descriptive and inferential statistical analysis was carried out. Chi-square/Fisher Exact tests were used to identify significance on categorical data and non-parametric settling was used for qualitative data analysis.

Results

A total of 365 patients were referred to the service from January 2016 to November 2017. 58% patients had grade I intestinal failure. 34% and 24% referrals were made by the colorectal and hepato-pancreato-biliary (HPB) units, respectively. Gut rest (27%) and small bowel obstruction (25%) were the most common indications for nutritional support. Duration of PN requirements varied greatly, but most patients (39%) required it for up to seven days. Single lumen PICC line (64%) and cephalic vein (54%) were the most commonly used catheter and site of vascular access, respectively. Chronicity of IF and number of vascular catheters required to provide PN were significantly associated with rates of CRS.

Conclusion

CRS was found to be statistically significantly associated with chronic IF and number of vascular access catheters required to provide PN. Cost implication and morbidity of CRS could be analysed further to identify other ways to provide safer and cost-efficient parental nutrition for patients with intestinal failure.

## Introduction

Intestinal failure (IF) was first defined in 1981 by Fleming and Remington as “a reduction in the functioning gut mass below the minimal amount necessary for adequate digestion and absorption of food” [1]. The aetiology of IF may be acquired or congenital, gastrointestinal or systemic, and benign or malignant and it occurs in all age groups [2]. IF can occur relatively abruptly, or may be the slow, progressive evolution of a chronic illness. It may either be a self-limiting short-term or a long-lasting condition [3]. Treatment of chronic intestinal failure (CIF) is based on providing intestinal rehabilitation with a target to restore intestinal function through nutrition, pharmacological and/or surgical therapy [4]. Those patients with irreversible intestinal failure will require long-term or lifelong home parenteral nutrition (HPN) or will need intestinal transplantation [5].

On the basis of onset, metabolic and expected outcome criteria, IF is classified as type I-III [5]. Type I is an acute, short-term and usually self-limiting condition. Type II is a prolonged acute condition, often seen in metabolically unstable patients, requiring complex multi-disciplinary care and intravenous supplementation over periods of weeks or months. Type III is a chronic condition, in metabolically stable patients, requiring intravenous supplementation over months or years, it may be reversible or irreversible.

The service provides specialist nutritional and Intestinal Failure Care to patients in the region and is one of the largest clinical Gastroenterology centres in the UK and provides a number of tertiary level gastroenterology and surgical services. In addition to receiving patients from within all clinical specialties in the trust the service takes referrals from other hospitals within the region.

Catheter-related sepsis (CRS) is defined as the presence of bacteraemia originating from an intravenous catheter. It is one of the most frequent, lethal, and costly complications of central venous catheterisation and also the most common cause of nosocomial bacteraemia. CRS is suggested by a temperature that begins 1/2 to 3 hours after the PN begins in those feeding at night only (cyclical PN) whereas in those fed continuously it is often a low-grade persistent temperature (British Association of Parenteral Nutrition, BAPEN) [5].

## Materials and methods

The data from the service from January 2016 to November 2017 was analysed retrospectively to understand and highlight the scope of services. BAPEN guidelines was used to diagnose CRS [5]. The findings of that study are illustrated in this paper. Descriptive and inferential statistical analysis was carried out in this study.

Results on continuous measurements are presented on mean and standard deviation (min-max) and the results on categorical measurements are presented in numbers (%). P value < 0.05 was considered statistically significant. The assumptions made on the data were that dependent variables should be normally distributed and that samples drawn from the population should be random, cases of the samples should be independent.

Chi-square/Fisher Exact test was used to find the significance of study parameters on categorical scale between two or more groups. Non-parametric setting was used for qualitative data analysis. Fisher’s exact test was used when cell samples were very small. P value < 0.05 was considered statistically significant. Statistical software namely SPSS version 18.0 (SPSS Inc., Chicago, IL), and R environment ver. 3.2.2 were used for the analysis of the data. Microsoft Word and Excel were used to generate graphs and tables.

## Results

During the 23-month period from January 2016 to November 2017, a total of 365 patients were assessed and managed for intestinal failure by the service across three hospital sites. Majority of the patients had grade I IF (58%), followed by grade II (26%) and grade III (12%). We were unable to get functional classification data for 14 patients, and they were excluded from rest of the study.

The highest number of referrals to the LIFT were made from the Colorectal units (34%) across both sites, followed by HPB (24%) and Gastroenterology (9%). Nearly half the referrals came from the hospital site at which the HPB unit was based.

The aetiology of IF can be classified into five major pathophysiological causes, such as short bowel, intestinal fistula, intestinal dysmotility, mechanical obstruction and extensive small bowel mucosal disease. Most causes of intestinal failure will fit into these major subtypes. Gut rest (26.8%), small bowel obstruction (25.1%) and ileus (15.4%) were the most common indications for referral for PN. These indications were together responsible for nearly 67% of all referrals. Other reasons for referral were build up for malnutrition, enterocutaneous fistulae, high output stomas and anastomotic leaks. The number of days PN was required for patients ranged from 1 to 178 days with a mean of 18.61 days.

Several different veins were used to gain vascular access for PN. The most preferred (54%) was the cephalic vein, followed by jugular vein (27%) and ante-cubital vein (11%). Various types of vascular catheters were used to provide PN; single lumen PICC lines (64%) were used most often. Vascular access catheters for PN were placed by various grades of professionals including registrars (39.3%), consultants (28.7%), nurses (27.9%), junior medical staff (0.9%). The grade of the professional securing the vascular access was not recorded for 11 (3.3%) patients.

There were several reasons for cessation of PN. The most common reason was successful initiation of enteral feeding (63.5%), followed by death (7.7%), home PN (6.6%), CRS (5.4%), transfer out of hospital trust (4.8%), pyrexia (4.3%) and withdrawal from treatment (3.4%). The reason for cessation of PN was unknown in 17 patients (4.8%). 82.3% of all patients managed to receive their quota of PN with the use of a single vascular line. 11.7% required two vascular lines, 6% required more than two vascular access lines for PN.

Twenty-three of 351 patients (6.5%) developed CRS during the study period. We tried to find any statistical correlation to identify the potential causes for CRS. We analysed nearly 10 such variables. We found that the grade of IF and the number of vascular access required to provide PN were statically significantly associated with (CRS) (Tables 1, 2). The following factors were not statistically significantly associated: age, gender, indication for PN, duration of PN, vascular access site, location and grade of professional gaining vascular access.

**Table 1:** Correlation between CRS and grade of IF P value = 0.034 Significant (Chi square test). IF: Intestinal failure

Grade of IF	Catheter-related sepsis (CRS)		Total
	No	Yes	
1	204 (96.2%)	8 (3.8%)	212
2	85 (89.5%)	10 (10.5%)	95
3	39 (88.6%)	5 (11.4%)	44
Total	328 (93.4%)	23 (6.6%)	351

**Table 2:** Correlation between CRS and number of vascular access lines required for PN P Value = 0.001 Significant (Chi square test). PN: Parenteral nutrition

Number of vascular access lines required to provide PN	Catheter-related sepsis (CRS)		Total
	No	Yes	
0	4 (100%)	0	4
1	270 (94.1%)	17 (5.9%)	287
2	41 (100%)	0	41
3	8 (72.7%)	3 (27.3%)	11
4	4 (80%)	1 (20%)	5
5	0	1 (100%)	1
6	1 (50%)	1 (50%)	2
Total	328 (93.4%)	23 (5.6%)	351

## Discussion

The scope of services provided was huge, serving nearly 360 patients in less than two years. Majority of the referrals for PN came from the colorectal and HPB surgical teams. Most patients requiring PN, expectedly had functional Grade I IF, and this is directly correlated to the number of referrals with bowel obstruction and those requiring gut rest. The duration of PN varied greatly, vast majority of patients requiring it for up to 15 days. Gut rest and intestinal obstruction were the most common indications for PN. Single lumen PICC line and the cephalic vein were the most preferred catheter and site to gain vascular access for PN.

The most common indication for the cessation of PN was the successful resumption of enteral feeding. Nearly three quarters of patients only need a single vascular access to receive their quota of PN. The chronicity of IF and increasing number of vascular access required to provide PN were statistically correlated with CRS in our study. The reasons for this could be that those patients with more chronic forms of IF were immunocompromised and more susceptible to sepsis. These patients were potentially hospitalised for long periods and were more likely to develop CRS.

Venous catheters used to provide PN become colonised with bacteria 24 hours following their placement. The exterior of these catheters become contaminated with skin commensals during placement and the luminal colonisation ensues, mostly from the catheter hub during connection and disconnection [6]. CRS is a major challenge for intestinal failure services and is a potentially life-threatening complication [7,8].

The dilemma faced by most professionals suspecting CRS is whether to lose a vascular access or risk life-threatening sepsis [6]. CRS in short-term catheters usually requires removal, however long-term catheters may be treated with a week of appropriate antibiotics given down the catheter [9]. The use of through catheter antibiotics can help rescue most catheters and prevent life-threatening sepsis [8]. Although a large body of this evidence comes from studies on children, there is no evidence to suggest it cannot be emulated in adults [10].

In patients requiring long-term PN, vascular access acts as a lifeline, however it is also the rate limiting step in providing long-term PN. Through catheter antibiotics, using dedicated PN only catheters, specialised training for staff using these catheters can help mitigate costs and associated morbidity and mortality. The cost implication, morbidity of CRS and prevention modalities could be studied further.

The limitations of this study were that this was a retrospective registry analysis. There were a few patients that were lost to follow up and were excluded from the study. This was also a non-interventional descriptive study and there was no investigational component to the study.

## Conclusions

Catheter-related sepsis is the biggest challenge facing intestinal failure services everywhere. We tried to identify causes for CRS in patients receiving parenteral nutrition. We studied various patient-related factors that could be linked to CRS. Only two of the parameters we studied were found to be statistically significant. We found that patients who had a higher grade of IF (II & III) were more likely to develop CRS. It also became evident that those patients who required repeated changes of the vascular access sites, were at higher risk for CRS. These outcomes helped us plan our services better. Being able to identify these risk factors might result in decreased costs for the services and better outcomes for the patients.
